# Clinical and Pathological Reports

**Published:** 1820-01-01

**Authors:** 


*Clinical and Pathological Reports.* By SAMUEL BLACK, M.D. of Newry. One volume Octavo, pp. 234. London, 1819.

NOTHING can be more true than the observation of Dr. Black, that "the physician who ascertains half a dozen of important *facts*, performs a more valuable, though a less splendid atchievement, than he who invents a dazzling theory." But we are, at the same time, to bear in mind, that the greatest theorists have, generally speaking, been the most accurate observers. We have only to take Cullen and Darwin, in our own country, for an exemplification of this remark. Chemistry has advanced more rapidly than medicine; and the *reason* of this, Dr. B. thinks, is because the *former* "has been cultivated entirely in the way of experiment and observation." But, in the first place, we are far from agreeing with our author, that the doctrines of chemistry are founded on an imperishable basis—

> —— "quod nec jovis ira nec ignis
> Nec poterit ferrum aut edax abolere vetustas."*

---

* As we have been visited with a pretty handsome share of censure from many quarters for the introduction of poetical quotations, and as the sins of others have been often laid upon our shoulders, we deem it necessary

And, in the second place, we are disposed to think, that an investigation of the laws which govern *dead* matter is a much more easy concern than that which bears reference to *living* and organized bodies, whether in health or disease. If, then, the sister sciences have advanced with *unequal* pace, we are quite confident that it is more owing to difference in the nature of the sciences themselves, than to any difference in the modes of their cultivation. We are, at the same time, well aware that the progress of medicine will be most accelerated "by the collection and arrangement of facts," because *data* are wanting in almost every branch of the science, and because every member may, and ought, to contribute his mite to the formation of this solid and essential substratum. The inferences and general laws which may ultimately grow out of this store of facts, men of genius only can manage.

From these considerations, Dr. Black has been induced to submit to the profession the clinical and pathological reports which constitute this volume; most of which, however, have already appeared as detached papers in the Transactions of different Societies. Several of them have been noticed in the Monthly and Quarterly Series of this Journal; and consequently, must be passed over in this place. The previously published papers are, on angina pectoris; dissections of two habitual drunkards; case of gouty affection; cases and dissections illustrative of disease of the brain. The papers hitherto unpublished are three—peritonitis chronica, otitis, and diabetes mellitus. These we shall proceed to analyze.

1. *Peritonitis Chronica.* Chronic inflammation of the peritonæum being not only an idiopathic disease, but a sequel of the acute form, is by no means of very rare occurrence. It is strange, therefore, that Dr. Cullen has no where mentioned it. Dr. Black apologises for designating by the term "*factitious membrane,*" what Dr. Baillie calls the "membrane of adhesions;" but Dr. B. is not singular or original in this. There is a very long and interesting paper on these factitious membranes in the 32d volume of the *Dict. des Sciences Med.* which we would recommend Dr. B. to consult. Two cases, illustrative of this dangerous and insidious disease, are related by Dr. Black; of

---

to state, that such poetical quotations as appear in this Journal are not ours, but those of the authors reviewed. It is to be hoped, that in these cases, we are not answerable for the deadly sins of all our contemporaries, as well as our own.

which we shall present a condensed analysis, with some observations of our own.

*Case* 1. A lady, ætat. 39, experienced a very tedious, severe, third labour, in August 1816, and two or three hours after delivery, was seized with acute pain over the whole abdomen, accompanied by exquisite tenderness and great tension. No alvine evacuations could be procured for eight days; during which, she had high fever, burning heat internally, excessive thirst, and severe head-ache. The fæces, when passed, were prodigious in quantity, fœtid, dry and indurated, and the evacuation attended with such excessive pain, that she attributed her delirium to this circumstance. Although a supervening diarrhœa relieved these symptoms, she was by no means free from abdominal pains, nor her health re-established till more than three months had elapsed. During this convalescence, her attention was excited to a severe pain in the right iliac region, which became permanent, and afterwards the seat of a supposed tumour. In January, 1818, she again became pregnant; and as the uterus expanded, she was tormented by pains in the pubic region, in the inguina, and by abdominal tenderness, to which were added tenesmus and micturition. In May, she had an attack of pyrexia, followed by uterine hæmorrhage, abortion, and extreme debility. Her recovery was tedious and imperfect; and ever afterwards she had acute lancinating pains through the abdomen, with irregular and profuse catamenia. Acidity, flatulence, nausea, with white tongue, prevailed the while, attended by obstinate constipation of the bowels

On the evening of the 25th of September 1818, she was seized, while walking in the garden, with rigors, and a degree of debility amounting nearly to syncope. She complained of severe pain in the abdomen, especially in the iliac region before mentioned.

"At this part, a considerable tumour could be distinctly felt, which, though it imparted to the fingers that crepitus which confined flatus gives, was yet exquisitely sensible, and impatient of pressure." P. 160.

In the month of October, a second tumour was tangible near the right groin, as large as an egg, and exquisitely sensible. It was now that Dr. Black was called in. On examining the larger tumour, he conceived it to be nearly as big as a small orange, and that he could encircle it within his grasp. Dr. B. observed that the patient exhibited that sallow and languid paleness, together with that cadaverous expression of countenance, so indicative of visceral disease, and which he considered beyond the reach of medicine. She continued to sink, had occasionally slight rigors, and often colliquative sweats. She died on the 6th of January, 1819.

*Dissection.* The omentum was an exceedingly large, dense, and massy viscus, in most places nearly half an inch thick, stretching from the great curvature of the arch of the colon, to which it was firmly adherent. Hence it extended to the pubes, where it adhered extensively to a great mass of factitious membrane, by which the uterus, bladder, and rectum were enveloped and confounded. It was vascular, and adherent to the concave surface of the liver, and in many places to the small intestines. From its interior surface oozed a purulent secretion. The intestines appeared unusually voluminous and ponderous; the mesentery thickened, with a purulent secretion on its surface; its glands black and shrivelled; the convolutions of the intestines universally bound together by great masses of factitious membrane of great thickness, solidity, and strength. Many portions of the intestinal tube were narrowed in their diameter, and bound down by bands of adhesive membrane. Marks of inflammation were visible throughout the whole abdominal cavity. The rectum was highly diseased, its coats thickened, and part of it sphacelated. The peritoneum lining the abdomen, phlogosed, and, in many places, thickened, opake, and inflexible. The cavity of the abdomen contained more than two quarts of a thin, turbid, *purulent* fluid, which must have been secreted from the inflamed surfaces, as there was no abscess to be found.

Dr. Black observes that this was clearly a *chronic* supervening on an *acute* peritoneal inflammation, and resulting from improper management, since

"A stimulant diet, with wine and other fermented drink, was used during the greater part of the period without the least reserve; whereas, frequent depletion of the vessels, both local and general; frequent small doses of calomel, perhaps combined with opium; the constant use of saline laxatives; warm fomentations to the abdomen; the tepid bath, and a milk diet, or at least a rigid antiphlogistic regimen, would have been requisite." P. 166.

In respect to the deceptive feeling of the tumours, we are not satisfied with Dr. Black's explanation. We are disposed to think, from some occurrences in our own practice, that these supposed tumours were inflated portions of intestine, when air was pent up from spasms or strictures, or both.

We shall introduce here a sketch of a case recently related by M. Gardien, in the *Dict. des Sciences Med.* which bears on the present subject.

"A trumpeter was wounded in the battle of Friedland by a sabre, the point of which entered beneath the false ribs of the right side. Five months afterwards he came under M. Gardien, worn down with sufferings and much jaundiced. While transporting to his Regimental Hospital in France, he experienced great pain from the least motion of the voiture, in the region of the liver. In 1808, however, he was able to rejoin his regiment in Spain, where, during three years, he complained of pain in the hepatic region, whenever his horse went to the trot. In 1811 he was again wounded in battle. The right hypochondrium, which was much bruised, became the immediate seat of a dull, yet pungent pain, which was greatly exasperated by every movement, even of the respiratory muscles. He could not lie on either side, but only on his back, the trunk gently bent forward. He had fever. Blood-letting, the antiphlogistic regimen, and other means were used at first; but the respiration remained laborious, at the end of three months, with dry cough, especially in the evenings and mornings. At this time he fell down stairs, pitching on his right side. Inflammatory symptoms were again lighted up, and he survived only eight days.

"*Dissection.* Right lung in a great measure hepatised [carnifié] the inferior portion of the pleura, on the same side, covered by a false membrane, completely organized, and binding the lung to the diaphragm. The liver adhered to the opposite surface of the abdomen by several columns of false membrane, and was covered, to a great extent, with an organized membrane three or four lines in thickness. The whole peritonæum was inflamed, and bathed in a reddish serous fluid, in which were floating shreds of coagulable lymph. All the peritoneal coverings of the abdominal viscera were coated with this falsemembrane, of recent formation."

*Case* 2. The subject of this case, a young lady, nine years of age, Dr. Black did not see till within a month of her death, though her disease had continued nearly eighteen months.

"I learnt, however, that the first symptoms of indisposition were great languor and listlessness, great disinclination to exertion of every kind, and a most unusual sensibility to cold. This was so remarkable, that when urged to walk out in clear cold weather, she was apt to return crying with violent pain in the abdomen. To this succeeded great fretfulness of temper, and an inability to draw up the trunk of the body perfectly straight without feeling pain in the abdomen. On the contrary, it was observed that she had an irresistable inclination to lean forward, so as to relax the parietes of the abdomen. It is to be observed that when a certain degree of emaciation was induced, an unfortunate plan was adopted of endeavouring to support the child's flesh and strength by bark, wine, and a stimulant regimen of animal food; and about six or seven months previous to her death, she was carried to the sea side, where she was frequently immersed in the cold bath—a ruinous practice!" P. 171.

When Dr. B. first saw this patient, she was weak and emaciated; confined to her bed; the pulse from 100 to 120; the appetite gone; the bowels costive; the abdomen tense and tumid, and tender.

"In two or three particular spots might be felt a circumscribed induration, accompanied with the most exquisite sensibility, which appeared to me to indicate the existence of some tumour underneath, or of some visceral obstruction. There occurred frequently through the day violent paroxysms of very acute pain in the abdomen, the duration of which was from three to five minutes, and which obliged the poor child to scream out most piteously." P. 172.

She died in a month after this period, reduced to the most extreme degree of emaciation and debility, and still harrassed with dreadful paroxysms of acute pain.

*Dissection.* On laying open the abdomen, no omentum could be seen. The peritoneal lining of this cavity was universally and highly inflamed; in many places thickened, opake, and inflexible. It was connected, at the caput coli, with the peritoneal coat of that intestine by several strong membranous bands; and this spot corresponded to the seat of the supposed tumour, which was exquisitely sensible and painful during life. The convolutions of the intestines were adherent by patches of false membrane of great density and firmness. In several places sacs were found, filled with pus. The mesenteric glands were all diseased, and when cut into contained curdy matter.

This, like the former case, would have required a strict antiphlogistic regimen; a rigid milk and vegetable diet; repeated local abstractions of blood; fomentations and counter-irritation to the surface of the abdomen; regular aperients, and perhaps a gradual and gentle introduction of mercury into the system, to arrest the progress of the adhesive inflammation. Perfect quietude, and the occasional warm bath, would have been serviceable. All these things were neglected, and a wrong course pursued.

Dr. Black next relates a melancholy case of inflammation of the ear proving fatal, the outline of which is as follows:

A young lady, aged twelve years, was roused from her sleep by a severe pain in the left ear, which continued, with great tenderness of the neighbouring parts, attended with occasional rigors and fever, but with very trifling discharge from the ear. Great debility came on, with intolerance of light or noise, and total inappetency. Leeches, purges, blisters, poultices, fomentations, venesection, calomel and antimony, procured no relief. At length a paralytic affection of some of the facial muscles supervened; the mouth was, at times, drawn to one side; the left palpebra was incapable of closing, so as to cover the globe of the eye; vision of the left eye was imperfect; pupils very large: in short, there was reason to fear a translation of disease to the membranes of the brain. In about a fortnight from the commencement of the disease, symptoms of thoracic inflammation appeared suddenly. The respiration became more and more disturbed, with cough, and acute pain in the chest, which seemed to cut her when she attempted to move, or even to take in her breath. Rigors, profuse perspiration, intense thirst, distressing paroxysms of coughing, great dyspnœa, and inability to lie down, harrassed this unfortunate young lady to the last, and she was relieved from her sufferings by the kind hand of Death, in less than a month from the date of the attack.

*Dissection.* All the parts of the internal ear were broken down into a mass of suppuration; but every thing within the cranium was perfectly sound and healthy. "The most attentive scrutiny could not discover any trace of organic disease or of effusion." In the thorax, indications of high inflammation were universal through the whole of this cavity, with considerable effusion of fluid, in which floated numerous filaments of coagulable lymph. Both lobes of the lungs were full of very small abscesses, apparently of recent formation.

The paralytic affections about the face and eyes, in this case, are explicable by the destructive process going on in the internal ear, affecting the portio dura of the auditory nerve, which communicates freely with branches of the fifth pair.

In respect to the thoracic inflammation, we would hardly call it a *translation*, but rather a supervention of disease from the great constitutional irritation occasioned by the otitis. The animal economy supplies extensive analogies in support of this presumption.

Dr. Black's volume closes with a case, and some observations on diabetes mellitus. We shall present an outline of the case, and then introduce a short paper of our own, which we had drawn up for another occasion.

A gentleman, 35 years of age, of very temperate habits, had been much exposed to alternations of heat and cold, in the super-intendance of a hat manufactory. His strength and voice began to fail him; his flesh wasted; his tongue was white; skin dry; thirst very distressing; urine in large quantity, and melleous on examination. An animal food diet, opium, and bleedings, ameliorated the symptoms, from time to time, and procrastinated his fate for nearly two years, when he died.

"*Dissection.* Mucous coat of the pylorus of a bright red colour, to the extent of some inches; veins of the intestines more turgid than usual; cortical substance of the kidnies not so firm as natural, but otherwise no morbid appearance. Vomicæ, abscesses, tubercles, and calculous concretions in the *lungs.* In the head, the arachnoid membrane separated from the pia mater throughout by a serous effusion; substance of the brain unusually firm; ten drachms of serous fluid in the ventricles."

We shall now introduce the following remarks on this deplorable malady.

Much discrepancy of opinion still obtains respecting the pathology of DIABETES, some attributing the disease to a *local* affection of the urinary organs, while others, with more reason, view it as an index of a more general constitutional affection—of a "break up of the constitution," or of a serious functional or organic derangement of some vital viscus, as the lungs or digestive apparatus. The following cases and dissections evince the unstable basis of diabetic pathology.

*Case* 1. A man, ætat. 32, apparently healthy, after having got wet in a storm, perceived that his stomach was out of order; that he wasted in flesh and strength; and that his visual powers were weakened. No defect was perceptible in the eye, except a slight dilatation of the pupils. The sight, however, diminished, until incomplete amaurosis was established. Diabetes mellitus had also advanced, *pari passu,* with the ocular complaint. Two pints of chalybeate water were ordered daily; and the patient retricted to animal food. The regimen, however, had hardly been entered upon, when the patient died suddenly, with but a slight and transient struggle.

On dissection, neither the optic nerve, brain, kidnies, or any other organ presented the slightest change of structure.*

This case shews us how *functional* disorder may destroy life, without leaving any trace of its existence on the dead tissues. We ourselves witnessed the dissection of a diabetic patient, a few years ago, where no organic change was perceptible in any viscus, during a minute investigation.

*Case* 2. A Dutch General, ætat. 45, of robust and sanguine temperament, suffered from chronic tarsal inflammation, apparently dependent on defective secretion of the lachrymal fluid. His thirst was constant; his mouth dry; his vital powers decreasing; bowels constipated; emaciation. The urea had totally disappeared from the urine, which was saccharine, clear, inodorous, and from sixteen to twenty pints in the day and night, while not more than half that quantity of fluid was drank.

The treatment was commenced with large doses of an "antiscorbutic syrup," which speedily brought out an eruption of small boils on the skin. Exclusive animal diet was enjoined. On the third day of this treatment, a faint yellow colour and some odour were apparent in the water, which was less saccharine. The morbid symptoms rapidly declined; the lachrymal secretion increased in proportion as the excess of urine was reduced; the functions of the digestive organs were re-established, and the patient was pronounced well in less than three months †

Very few dissections of diabetic patients are on record. In the *Dict. des Sciences Med.* M. Renauldin, after stating Dr. Baillie's minutes of a dissection, relates the following particulars of the examination of a diabetic body, by M. M. Dupuytren and Thenard. Stomach greatly enlarged in

* Journal Universel, &c. Avril, 1819.

† Dr. Krimer, a physician of Halle, has lately made a great number of experiments on animals, with a view of elucidating the nature of diabetes mellitus, and come to the conclusion that, in most instances, the disease depends on, or is connected with a deranged state of the biliary secretion. He ascertained, that in those hepatic complaints, where *bile* presented itself in the urine, the *urea* immediately disappeared, and the urine, in fact, became of a diabetic nature. He observes, Berzelius discovered that the bile, in health, contained a saccharine principle, and hazards a conjecture that when the liver fails to perform its proper function, a morbid vicarious action of forming sugar may be thrown upon the kidnies. He has proved, that when dogs are fed on rice or rye, the uric acid disappears from the urine, and they become diabetic, but are quickly cured by galvanism. Rev.

capacity, its internal surface covered with a network of dilated vessels. Duodenum and part of the jejunum and cæcum redder and thicker than usual. The kidnies one-third larger than natural; their structure somewhat soft and grey, but not more than usually permeable by injection. The urinary apparatus healthy in structure in all other respects. The right lung contained several small purulent collections; the left, some cysts filled with an elastic fluid, "remplis par fluide elastique." The muscles, as in most chronic diseases, pale and wasted.

From the great French work alluded to, we shall present the following therapeutical sketch, for the benefit of the English reader.

The ancient physicians having no chemical knowledge of the urine in diabetes, viewed the disease in the light of a morbidly excessive discharge merely; and hence their therapeutics were vague and inefficacious. It is curious, however, that the compilator, ÆTIUS, is the only one of the ancients, who made a remarkable approach to the best mode of treating the disease: namely, by ordering the patient to live principally on animal food, especially pork, and to drink good wine. Houllier, long ago, recommended blood-letting in diabetes. Rollo, Nicolas, Gueudeville, Dupuytren, and Thenard have proved that fat animal food, quietude, and total abstinence from vegetables, are the best remedial measures in this deplorable malady. For drink, wine and water should be preferred; occasionally milk.

When the animal food disagrees, or appears inefficacious, we must have recourse to medicine. Nicolas and Gueudeville have employed opium with success, in combination with cinchona. As for the long catalogue of boasted remedies, including Dover's powder, lytta, lime water, mercury, &c. there is little dependence to be placed on any of them. It ought to be borne in mind, however, that the lungs or digestive organs are generally in fault, and without correcting the derangements of these viscera, no cure can be expected.

It does not appear that our continental brethren have given any trial to *venesection,* so much employed in diabetic complaints of this country.

---

To Dr. Black the profession is much indebted for the many valuable cases and judicious observations contained in this volume. A true zeal for the improvement of medical science is conspicuous throughout the work, which is characterized by classic taste, sound judgment, and extensive information.

